# Shared and distinct functions of the pseudokinase CORYNE (CRN) in shoot and root stem cell maintenance of Arabidopsis

**DOI:** 10.1093/jxb/erw207

**Published:** 2016-05-26

**Authors:** Marc Somssich, Andrea Bleckmann, Rüdiger Simon

**Affiliations:** Institute for Developmental Genetics and Cluster of Excellence on Plant Sciences, Heinrich Heine University, Universitätsstr. 1, D-40225 Düsseldorf, Germany

**Keywords:** *Arabidopsis thaliana*, CORYNE, CLAVATA, pseudokinase, peptide signaling, stem cell maintenance.

## Abstract

Stem cell maintenance in plants depends on the activity of small secreted signaling peptides of the CLAVATA3/EMBRYO SURROUNDING REGION (CLE) family, which, in the shoot, act through at least three kinds of receptor complexes, CLAVATA1 (CLV1) homomers, CLAVATA2 (CLV2) / CORYNE (CRN) heteromers, and CLV1/CLV2/CRN multimers. In the root, the CLV2/CRN receptor complexes function in the proximal meristem to transmit signals from the CLE peptide CLE40. While CLV1 consists of an extracellular receptor domain and an intracellular kinase domain, CLV2, a leucine-rich repeat (LRR) receptor-like protein, and CRN, a protein kinase, have to interact to form a receptor–kinase complex. The kinase domain of CRN has been reported to be catalytically inactive, and it is not yet known how the CLV2/CRN complex can relay the perceived signal into the cells, and whether the kinase domain is necessary for signal transduction at all. In this study we show that the kinase domain of CRN is actively involved in CLV3 signal transduction in the shoot apical meristem of Arabidopsis, but it is dispensable for CRN protein function in root meristem maintenance. Hence, we provide an example of a catalytically inactive pseudokinase that is involved in two homologous pathways, but functions in distinctively different ways in each of them.

## Highlight

This study shows that the pseudokinase domain of the CLV2 co-receptor CRN is actively involved in transmitting the CLV3 peptide signal in the Arabidopsis shoot, but not in the proximal root meristem.

## Introduction

In *Arabidopsis thaliana* all tissues are derived from the activities of the stem cell populations of the meristem (Nägeli, 1858). Stem cell homeostasis in the shoot apical meristem (SAM) depends on the activity of the CLAVATA (CLV)–WUSCHEL (WUS) negative feedback loop (Brand *et al.*, 2000; Schoof *et al.*, 2000). WUS, a homeodomain transcription factor, is expressed in a small group of cells in the rib meristem, residing beneath the apical stem cell domain of the SAM (Laux *et al.*, 1996; Mayer *et al.*, 1998). The WUS protein has been shown to move upwards from its expression domain into the central zone and promote stem cell fate, and also the expression of the small secreted peptide CLAVATA3 (CLV3) (Brand *et al.*, 2000; Yadav *et al.*, 2011; Daum *et al.*, 2014). CLV3 signals from the stem cell domain mainly through the receptors CLAVATA1 (CLV1), CLAVATA2 (CLV2) and CORYNE (CRN) to restrict *WUS* expression, thus establishing a negative feedback loop (Brand *et al.*, 2000; Schoof *et al.*, 2000). Plants carrying mutations in *CLV1*, *CLV2*, or *CRN* generate more stem cells due to less restricted *WUS* expression, which results in a larger meristem that subsequently generates more vegetative or floral organs (Clark *et al.*, 1993; Kayes and Clark, 1998; Müller *et al.*, 2008). Utilizing organ number as a phenotypic indicator allows signaling activity to be quantified indirectly, and studies using single- and double-mutant combinations have indicated that CLV1, and CLV2 together with CRN, act in two parallel pathways to regulate stem cell fate (Müller *et al.*, 2008). Specifically, *clv3* mutants exhibit the strongest phenotype (increase in carpel number), while *clv1*, *clv2*, or *crn* single-mutants show only intermediate phenotypes (Clark *et al.*, 1993, 1995; Kayes and Clark, 1998; Müller *et al.*, 2008). The *clv2*/*crn* double-mutant resembles the respective single-mutants, genetically positioning CLV2 and CRN in the same pathway (Müller *et al.*, 2008). *clv1*/*clv2* or *clv1*/*crn* double-mutants, however, show additive phenotypes, which are comparable in strength to the *clv3* mutant (Kayes and Clark, 1998; Müller *et al.*, 2008). Accordingly, CLV1 was thought to act independently from and in parallel to CLV2/CRN (Müller *et al.*, 2008). This is further supported by the molecular properties of these proteins. All three receptors localize to the plasma membrane (PM). *CLV1* encodes a leucine-rich repeat (LRR) receptor-like kinase (RLK) with an extracellular LRR receptor domain, a transmembrane domain (TMD), and an intracellular kinase domain (KD) (Clark *et al.*, 1997). CLV2 carries an extracellular LRR receptor domain and a TMD, but lacks an intracellular KD (Jeong *et al.*, 1999). *CRN* encodes a kinase protein carrying a TMD and lacks a large extracellular receptor domain (Müller *et al.*, 2008). CLV1 binds the CLV3 peptide and could therefore perceive and transmit the CLV3 signal by itself. CLV2 and CRN interact molecularly to establish a potentially functional receptor kinase, which has been shown by live cell imaging combined with Förster (Fluorescence) Resonance Energy Transfer (FRET) experiments (Bleckmann *et al.*, 2010). The formation of such CLV2/CRN heteromers was found to be a prerequisite for their export from the endoplasmic reticulum (ER) to the PM (Bleckmann *et al.*, 2010). CLV1 localizes independently of CLV2 and CRN to the PM and preferentially forms homomers. In addition, all three receptors can also interact to form a larger heteromeric complex, where the interaction between CLV1 and the CLV2/CRN heteromer is mediated by CRN (Bleckmann *et al.*, 2010). Furthermore, higher levels of CLV3 peptide induce the formation of these larger multimers and their subsequent relocation into specific PM subdomains (Somssich *et al.*, 2015). This has led to the hypothesis that the CLV1 homomers and CLV2/CRN heteromers act in two independent signaling pathways that can simultaneously be down-regulated through sequestration of the receptors in PM subdomains (Bleckmann *et al.*, 2010; Somssich *et al.*, 2015).

The notion that CLV1 and CLV2/CRN function at least in part independently to transmit the CLV3 signal implies that both CLV1 and CLV2 can (directly or indirectly) perceive the CLV3 peptide, and that the cytoplasmic kinase domains of CLV1 and CRN transmit the signal to downstream effectors. Consistent with this, the CLV1 ectodomain was found to bind the CLV3 peptide and its intracellular kinase autophosphorylates after treatment with the peptide (Stone *et al.*, 1998; Ogawa *et al.*, 2008; Shinohara and Matsubayashi, 2015). However, although CLV2 can potentially bind a variety of related CLE peptides, it has no increased specificity for binding CLV3 compared to other CLE peptides (Guo *et al.*, 2010; Shinohara and Matsubayashi, 2015). Furthermore, the CRN kinase does not autophosphorylate following CLV3 treatment, although it should be noted that this *in vitro* experiment was performed without co-expression of the CLV2 receptor (Nimchuk *et al.*, 2011). Interestingly, the KD of CRN contains several features that are typical for signaling-inactive pseudokinases: it lacks the essential aspartic acid in the HRD sequence motif of the catalytic loop and one of the three conserved glycines in the G-loop (Nimchuk *et al.*, 2011). These two motifs are necessary for ATP binding. In addition, the DFG motif in the Mg^2+^ binding loop is not fully conserved, and the activation segment is truncated compared to active kinases (Nimchuk *et al.*, 2011). Based on these observations, it has been questioned whether the CRN kinase domain is actively involved in transmitting the CLV3 signal into the cell, and it has been suggested that CRN acts only as a scaffolding protein to localize CLV2 to the PM and mediate its interaction with CLV1 (Nimchuk *et al.*, 2011). To gain more insights into the role of CRN, we constructed a series of CRN protein variants in order to probe the different domains of the protein for their roles in mediating CLV2 interactions, promoting ER-to-PM export, and the contribution to CLE signaling pathways in shoot and root stem cell maintenance.

Here, we describe two different modes of action for CRN in shoot and root meristem maintenance in Arabidopsis. We find that the pseudokinase domain of CRN is essential for its function in CLV3 signaling in the shoot meristem, but not for the CLV1-independent signaling pathway through which CRN and CLV2 control root meristem homeostasis. Thus, CRN assumes both shared but also distinct functions in these two homologous pathways.

## Materials and methods

### *Arabidopsis thaliana* plant lines

The following plant lines were used: Landsberg *erecta* (L*er*), *coryne-1* (*crn-1*) in L*er* background (Müller *et al.*, 2008), Columbia-*0* (Col-*0*), *crn-3* and *clavata2-gk* (*clv2-gk*) (Rosso *et al.*, 2003) in Col-*0* background. *Agrobacterium*-mediated transformation was performed according to Logemann *et al.* (2006). To analyze the carpel phenotype, *Arabidopsis thaliana* plants were grown at 21 °C in short-day (8h light) conditions for 4 weeks and were then shifted to 21 °C continuous light conditions for 2 weeks, at which point the carpels were counted. For each variant, between 2 and 11 independent transgenic lines were analyzed. For peptide treatments, plants were grown on GM plates supplemented with 500nM CLV3 (RTV[Hyp]SG[Hyp]DPLHHH) at 21 °C continuous light conditions, and root length was measured 7 d post-germination using ImageJ.

### *Nicotiana benthamiana* plant lines

*Nicotiana benthamiana* plants were grown in the greenhouse for 4 weeks prior to transient transformation. Transformation and expression were as described previously by Bleckmann *et al.* (2010).

### Construction of plasmids

The different CRN and CLV2 variants for stable plant transformation were created from cDNA using Gateway^®^ BP / LR Clonase^®^ II Cloning Kits, as well as the destination vector pMDC32 described previously by Curtis and Grossniklaus (2003).

The inducible CRN and CLV2 green fluorescent protein (GFP) and mCherry expression vectors have been described previously by Bleckmann *et al.* (2010). The inducible Cerulean expression vector (pABindCerulean) was created based on pMDC7 (Curtis and Grossniklaus, 2003). The fluorophore fusions for the different CRN and CLV2 variants were created from cDNA using Gateway^®^ BP / LR Clonase^®^ II Cloning Kits, as well as the destination vectors pABindGFP and pABindmCherry described previously by Bleckmann *et al.* (2010).

The oligonucleotides used are shown in Table 1.

**Table 1.** Oligonucleotides used in the construction of plasmids

**Sequence**	**Forward**	**Reverse**
CRN	CACCATGAAGCAAAGAAGAAGA	AAAGCTGTGCAGTTGTGT
CRN promoter	AAAAAGCAGGCTAAAGATGCATAGGCTTGC	AAGAAAGCTGGGTGCTGCTTCTACGAATAA
CRΔEC	ACAAGCACAAGTACAAGTGTTATTGTGATTAGTATC	AAAGCTGTGCAGTTGTGT
CRΔEC1	AAGTACAAGTTGTGCAGCAGCAACTGTTGCACACTTATCC	GGATAAGTGTGCAACAGTTGCTGCTGCACAACTTGTACTT
CRΔEC2	ACACCACTTGAATCAGCAATCACTTCCAAGGTTATTGTGA	TCACAATAACCTTGGAAGTGATTGCTGATTCAAGTGGTGT
crn-3	CACCATGAAGCAAAGAAGAAGA	AGAGGACTCAGGGGCAGAGTAG
CRΔKi	CACCATGAAGCAAAGAAGAAGA	AAGAAAGCTGGGTTGCTACGAACCAAGAAAGC
CR(SD)	GAAGGAGTTGGTGACCCAGAAAGTAGT	ACTACTTTCTGGGTCACCAACTCCTTC
CR(SA)	TCACTTGAAGGAGTTGGTGCACCAGAAAGTAGTAGT	ACTACTACTTTCTGGTGCACCAACTCCTTCAAGTGA
CR (<>C1Ki)	CAGCTTTGGTGTTAGCTTTCTTGGTTC	GAACGCGATCAAGTTCGCCAC
CLV2	AAAAAGCAGGCTATGATAAAGATTGCAG	AAGAAAGCTGGGTAAGCTTTGGTCTGAAGAA
CLV2 promoter	CACCAGACACAAAGCCCTTTCCATTGTC	CTTTATCATAGCTCAGAGGA
C2(RA)	CACCATGATAAAGATTGCAGAT	AGCTTTGGTCTGAAGAATATAACTAGCAGCACGAG
C2(708)	CACCATGATAAAGATTGCAGAT	TGAGCAAAAGATACCTAACA
C2(CRKi)	TTAGCTTCGATTTTGGAGTGTTAGGTCGTAGCATTGTCAAATTCATGAAA	TTTCATGAATTTGACAATGCTACGACCTAACACTCCAAAATCGAAGCTAA

### Microscopy

Imaging and FRET-APB (acceptor photobleaching) measurements were done on a Zeiss LSM 780 confocal microscope. GFP was excited with a continuous wave 488nm argon laser at the objective (40× water immersion, Zeiss C-Apochromat 40×/1.20W korr M27) and emission was detected at 499–517nm by 32-Channel-GaAsP-Detectors. mCherry was excited using a 561nm continuous wave diode laser and emission detected at 595–635nm. Cerulean was excited with a continuous wave 458nm argon laser at the objective (40× water immersion, Zeiss C-Apochromat 40×/1.20W korr M27) and emission was detected at 459–485nm by 32-Channel-GaAsP-Detectors. For APB measurements, a series of 12 256×256 pixel frames with 0.18 µM pixel size, 47 µm^2^ image size and 1.27 µs pixel dwell time was recorded. After five frames, mCherry was photobleached in a region of interest along the PM by 80 iterations with 100% laser power. Donor-dequenching (in %) was determined as GFP intensity change after photobleaching as follows: (GFPafter − GFPbefore)/GFPafter × 100.

## Results

### Expression patterns of CLV2 and CRN in the proximal root meristem

**Fig. 1.**
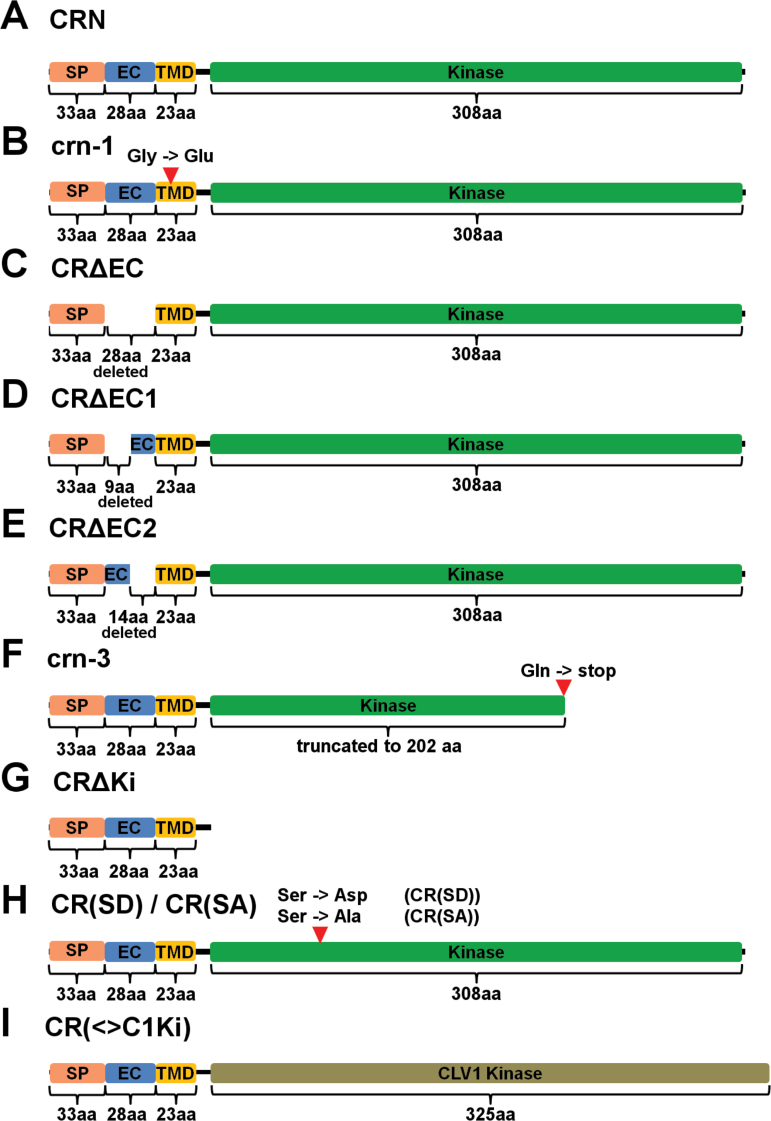
Schematic representations of the CRN protein variants. (A) CRN (wild-type); (B) crn-1; (C) CRΔEC; (D) CRΔEC1; (E) CRΔEC2; (F) crn-3; (G) CRΔKi; (H) CR(SD) and CR(SA); (I) CR (<>C1Ki). Light orange = signal peptide (SP); Blue = extracellular domain (EC); Yellow = transmembrane domain (TMD); Green = kinase domain; Olive = CLV1 kinase domain. aa = amino acids. The red arrowheads in (B), (F) and (H) indicate the positions of amino acid exchanges.

**Fig. 2.**
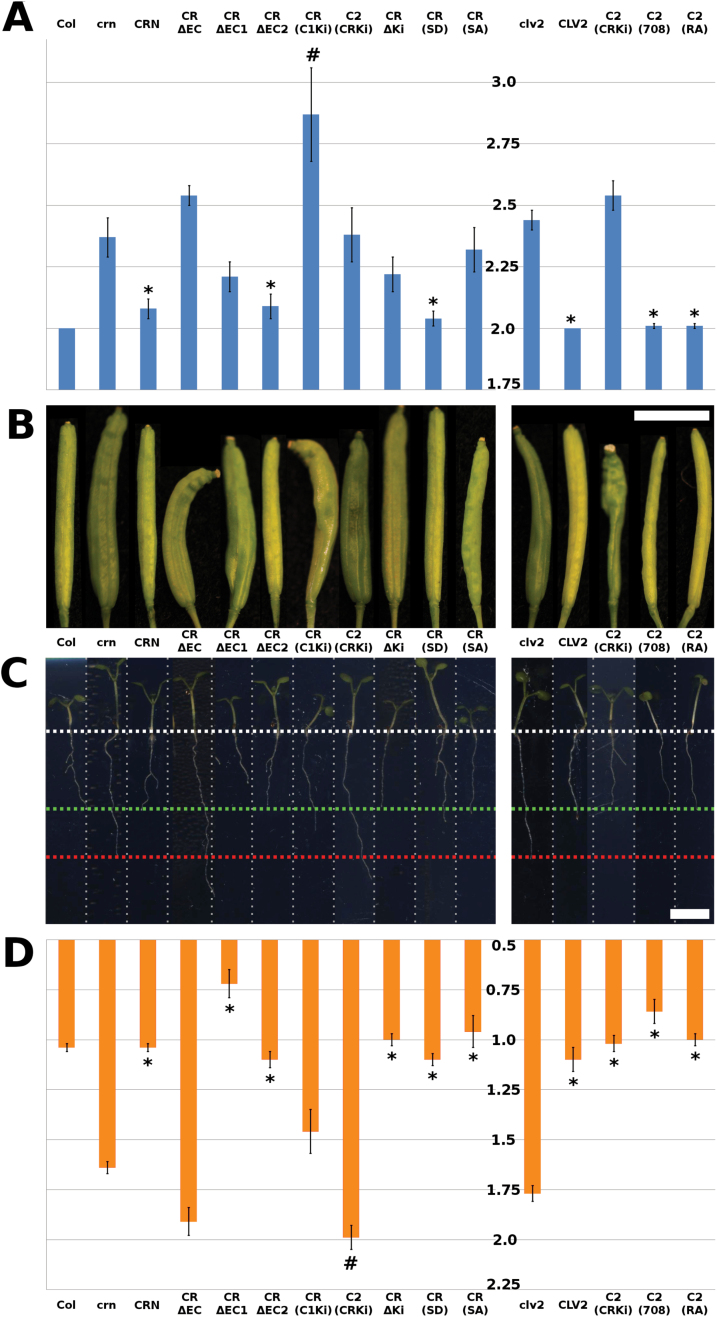
Carpel and root phenotypes of *crn* and *clv2* mutants. (A) Quantification of the carpel phenotype. The scale (1.75–3.0) shows the number of carpels per silique in the different lines. (B, C) Representative siliques (B) and seedlings (C) of the different transgenic and reference lines. The seedlings in the root assay were grown on GM-medium containing 500nM CLV3 peptide. The reference lengths are indicated by the dotted lines: white = start of root; green = wild-type; red = mutant (*crn* or *clv2*). For the CRN(SA) variant, three independent transgenic lines were analyzed. For all other variants, between 7 and 11 independent transgenic lines were analyzed. Between 10 and 15 siliques per plant were analyzed from at least five different plants from each line. (D) Quantification of the root phenotype. The scale (0.5–2.25) is root length in cm. The different lines are: Col = Wild-type; *crn* = *crn*-3; *clv2* = *clv2-gk*. The following variants are expressed in the *crn-3* background: CRN, CRΔEC, CRΔEC1, CRΔEC2, CR(C1Ki), C2(CRKi), CRΔKi, CR(SD), and CR(SA). The following variants are expressed in the *clv2-gk* background: CLV2, C2(CRKi), C2(708), and C2(RA). * Indicates values significantly different from the mutant. ^#^ Indicates an enhanced mutant phenotype significantly different from the mutant. Bars in the graphs are standard error. Scale bars in the images are 0.5cm. For the CRN(SA) variant, two independent transgenic lines were analyzed. For all other variants, between 7 and 11 independent transgenic lines were analyzed. Between 33 and 122 roots were measured from each line.

Increased *CLV3* expression results in the termination of meristematic activity due to differentiation of stem cells. The *crn-1* allele was identified in an ethylmethanesulfonate (EMS) mutagenesis screen as a suppressor of this meristem arrest phenotype in a *CLV3*-overexpressing (ox) *Arabidopsis* Landsberg *erecta* (L*er*) line (Müller *et al.*, 2008). The mutation in *crn-1* results in an amino acid exchange G70E in the predicted TMD of the CRN protein (Fig. 1B, Supplementary Fig. S1C at *JXB* online) (Müller *et al.*, 2008). From its protein sequence, the TMD of CRN is expected to form a β-barrel, and the structurally small glycines (G) are proposed to provide the flexibility needed to form these (Hutchinson and Thornton, 1994). Therefore, replacing the uncharged glycines with a negatively charged and structurally larger glutamic acid (E) could interfere with β-barrel formation and thereby PM integration of the protein and interaction with CLV1 and CLV2, resulting in the *crn-1* mutant phenotype (Müller *et al.*, 2008; Bleckmann *et al.*, 2010). Mutations of the *clv*-signaling pathway cause stem cell accumulation, which results in the formation of larger floral meristems, production of additional carpels, and generation of an aberrant silique. *crn-1* mutant flowers produced 3.60 (±0.34) carpels on average, while the L*er* wild-type typically produced 2.04 (±0.05) (Müller *et al.*, 2008). A second available mutant *crn* allele is *crn-3*, which is in the Columbia-*0* (Col) background. This line was identified as an EMS-induced mutant in the *Arabidopsis* TILLING project and subsequently backcrossed three times to clean-up the genetic background for any additional mutations (Till *et al.*, 2003). *clv* mutants in the Col background typically exhibit weaker phenotypes compared to alleles in the L*er* background (Müller *et al.*, 2008). We found that *crn-3* mutant flowers produced 2.37 (±0.08) carpels on average, compared to 2.00 (±0) in Col (Fig. 2A, B). *crn-3* plants carry a point mutation at Q296, which causes a translational stop resulting in a shortening of the KD by one third (Fig. 1F, Supplementary Fig. S1E).

**Fig. 3.**
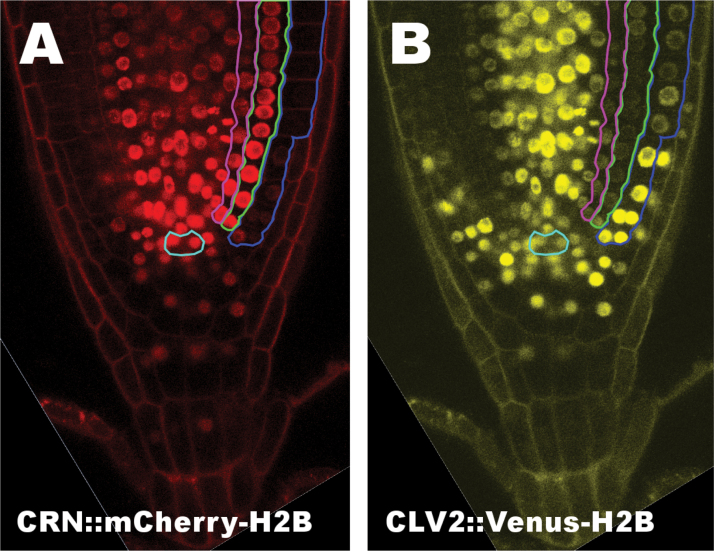
*CLV2* and *CRN* expressions patterns in the root of Arabidopsis.*CRN::mCherry-H2B* (A) and *CLV2::Venus-H2B* (B) expression in a Col-*0* root. For better differentiation between the tissues, the QC is encircled in turquoise. The epidermis is encircled in blue, the cortex in green, and the endodermis in pink in the right half of the roots.

In addition to their well-described roles in SAM homeostasis, CLV2 and CRN also play a role in root meristem maintenance. Overexpression or external addition of diverse CLE peptides, including CLV3, has been previously shown to restrict root meristem size (Hobe *et al.*, 2003; Fiers *et al.*, 2005). This signaling pathway, although not fully understood, requires the activity of both CRN and CLV2 (but not CLV1), because both *clv2* and *crn* mutants appear to be fully resistant against the root-shortening effect of CLE peptides (Hobe *et al.*, 2003; Fiers *et al.*, 2005; Pallakies and Simon, 2014). To further investigate the regulatory role of CRN and CLV2 in root meristem activity, we first determined the expression patterns of both genes in the root of Arabidopsis by employing fluorescence reporters (mCherry or Venus) fused to Histone 2B (H2B) for nuclear targeting. As regulatory sequences we used 1711bp upstream of the *ATG* for *CRN* and 1315bp upstream of the *ATG* for *CLV2*. To determine if these sequences contain all the regulatory elements necessary for wild-type *CRN* and *CLV2* expression, we tested their ability to complement the *crn-1* or *clv2-1* mutants when used in *CRN::CRN-GFP* or *CLV2::CLV2-GFP* constructs. Both were able to complement the mutants in all the transgenic lines obtained (Supplementary Fig. S2). We then generated transgenic Col plants coexpressing *CRN::mCherry-H2B* and *CLV2::Venus-H2B*. *CRN* promoted expression of the reporter in the stele, endodermis and cortex cells, and the QC with surrounding initials and columella cells, with an apparent expression maximum in the cells of the QC and the proximal initials (Fig. 3A). Expression was absent in mature epidermis and lateral root cap cells, and was only faint in the distal part of the meristem (Fig. 3A). *CLV2* expression was strongest in the stele and young epidermis cells, while it was only weak in endodermis, cortex and differentiated columella cells (Fig. 3B). Interestingly, while *CRN* expression appeared to be strongest in the QC and proximal initials, *CLV2* expression appeared weaker in those cells compared to the surrounding tissues (Fig. 3B). Furthermore, while *CRN* expression is strong in cortex and endodermis, *CLV2* is only weakly expressed there but is strong in the epidermis. Based on these expression patterns, it is possible that CLV2 and CRN could also act in concert in the proximal root meristem in a CLE-dependent fashion to regulate root meristem size, similar to their joint role in shoot meristem maintenance. Interestingly, however, their expression pattern does not completely overlap outside of the stem cell niche. Therefore, CLV2 might also have additional, CRN-independent functions in the epidermis. Furthermore, *CLV2* was also expressed distal to the QC in the columella cell initials and the cells lateral to them in a pattern similar to the expression pattern of *CLV1* in the distal root meristem (Stahl *et al.*, 2013).

### The extracellular domain of CRN is necessary to stabilize the CRN/CLV2 complex

**Fig. 4.**
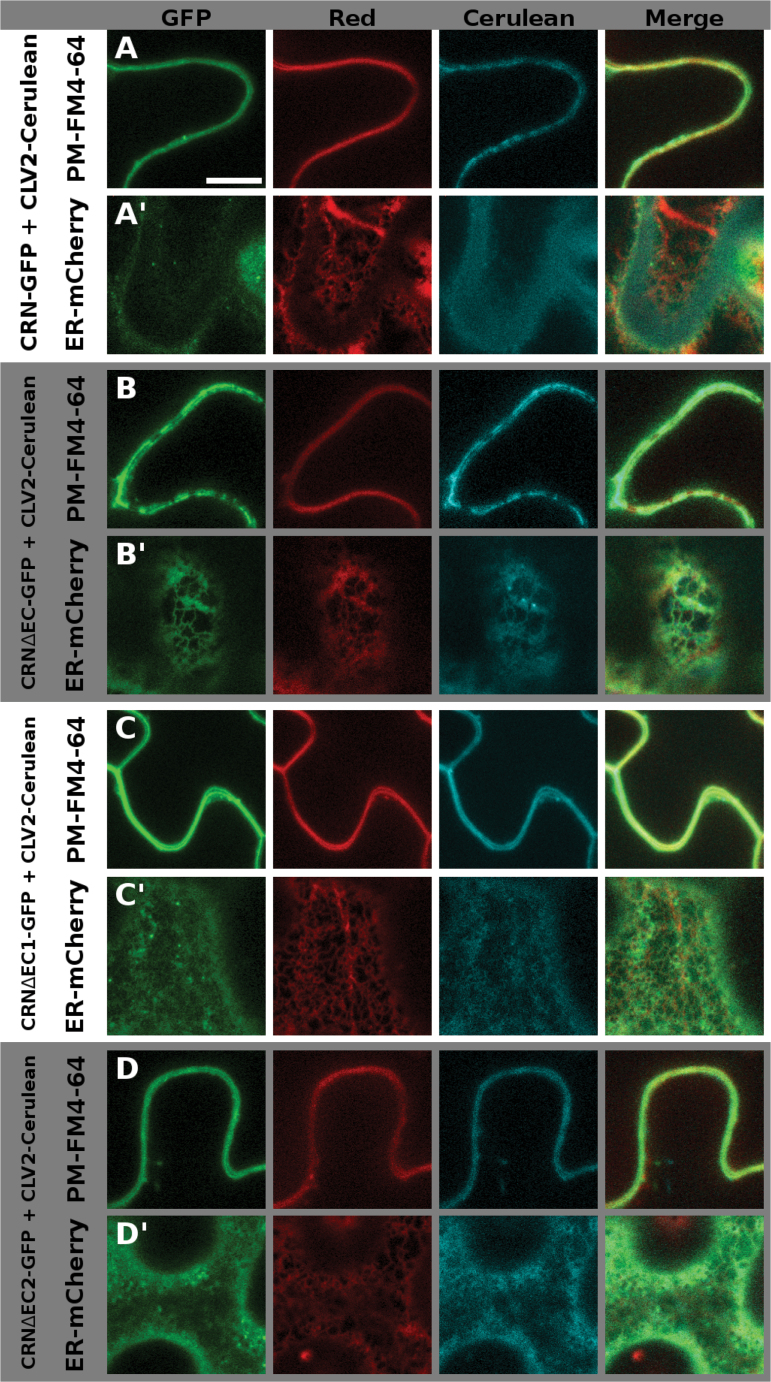
Intracellular localization of the different CRN ECD variants. The different CRN variants are tagged with GFP, and CLV2 is tagged with Cerulean. In the red channel, either the membrane dye FM4-64 (A–D) or ER-localized RTNLB2-mCherry (A′–D′) is shown. Wild-type CRN is shown in (A) and (A′), CRΔEC in (B) / (B′), CRΔEC1 in (C) / (C′), and CRΔEC2 in (D) / (D′). In the PM focal plane (A–D), the signal is homogenous along the PM for CRN, CRΔEC1, and CRΔEC2, co-localizing with FM4-64 (A, C, D), and patchy along the PM for CRΔEC (B). In the ER focal plane (A′-D′), there is signal visible in all CRN variants tested; however, the pattern is distinct from the net-like ER-structure exhibited by RTNLB2-mCherry in (A′), (C′), and (D′). Supplementary Fig. S4 shows the same cells with switched focal planes: the FM4-64 marked cell is focused on the ER, the RTNLB2-marked cell is focused on the PM. Scale bar = 10 µm.

**Fig. 5.**
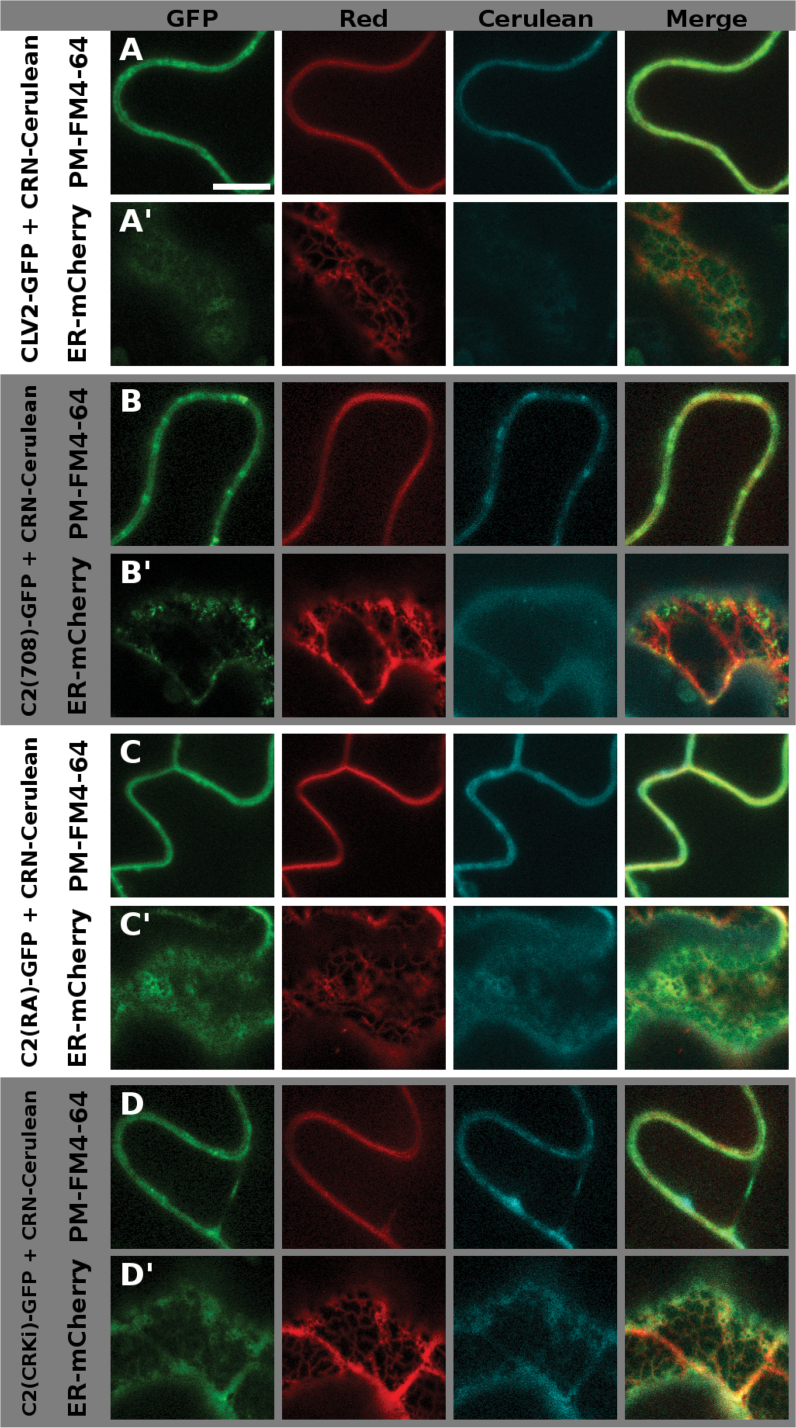
Intracellular localization of the different CLV2 variants. The different CLV2 variants are tagged with GFP, and CRN is tagged with Cerulean. In the red channel, either the membrane dye FM4-64 (A–D) or ER-localized RTNLB2-mCherry (A′–D′) is shown. Wild-type CLV2 is shown in (A) / (A′), C2(708) in (B) / (B′), C2(RA) in (C), (C′) and C2(CRKi) in (D) / (D′). In the PM focal plane (A–D), all four protein variants co-localize with FM4-64 (A–D). In the ER focal plane (A′-D′), there is signal visible in all CLV2 variants tested, but different from the distinct net-like ER-structure exhibited by RTNLB2-mCherry. This net-like structure is also visible in the C2(CRKi) protein (D′), indicating impaired, but not completely blocked ER-export of this CLV2 variant. Supplementary Fig. S5 shows the same cells with switched focal planes: the FM4-64 marked cell is focused on the ER, the RTNLB2-marked cell is focused on the PM. Scale bar = 10 µm.

**Fig. 6.**
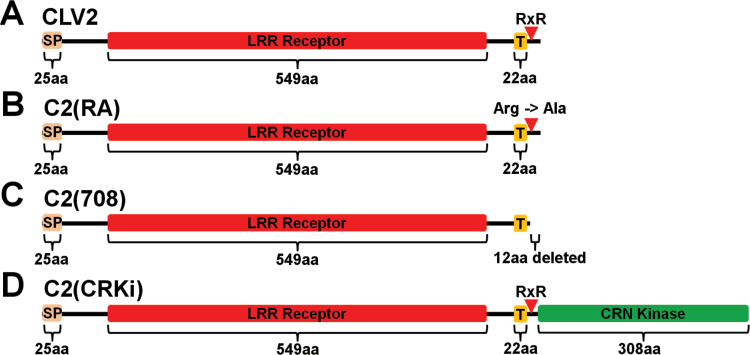
Schematic representations of the CLV2 protein variants. (A) CLV2, (B) C2(RA), (C) C2(708), (D) C2(CRKi). Light orange = signal peptide (SP); Red = LRR receptor domain; Yellow = transmembrane domain (T); Green = CRN kinase domain. aa = amino acids. The red arrowhead indicates the position of the putative RxR motif.

So far it is not understood how ER retention of the single CRN or CLV2 proteins and PM transport following heterodimerization are regulated. We tried to further elucidate this by analyzing sequences of the extracellular domain of CRN and the juxtamembrane domain of CLV2 for their role in this regard. To determine their ability to interact with CLV2 and promote ER-to-PM export of the formed complex, we transiently expressed fluorophore-tagged versions of the proteins in *Nicotiana benthamiana* and analyzed their intracellular localization, as well as their interactions via FRET measurements as described previously (Bleckmann *et al.*, 2010). To visualize the intracellular localization we used N-(3-triethylammoniumpropyl)-4-(p-diethylaminophenyl-hexatrienyl) pyridinium dibromide (FM 4–64) to stain the PM, and Reticulon-Like Protein subfamily B 2 (RTNLB2) tagged with mCherry or Cerulean to mark the ER (Vida and Emr, 1995; Sparkes *et al.*, 2010). We present two focal planes of the same cell for all expressed variants: first, the focus is on the PM (Supplementary Fig. S3). Here, the FM4-64 dye shows a homogenous distribution, whereas RTNLB2-Cerulean exhibits a patchy pattern that represents the fusion points of the PM with the ER. The second focal plane shows the inner region of the cell and the ER (Supplementary Fig. S4). Here, RTNLB-Cerulean exhibits a net-like structure, while the signal coming from FM4-64 is blurry, stemming from the out-of-focus PM. When we analyzed any of the CRN variants in the absence of CLV2, the protein was not exported from the ER where it co-localized with RTNLB2-Cerulean, displaying either the patchy pattern along the PM, or the net-like structure of the ER, depending on the focal plane (Supplementary Figs S3A, S4A). The same is true for CLV2 variants expressed without CRN (Supplementary Figs S3J, S4J). When co-expressing CRN-GFP and CLV2-Cerulean (or CLV2-GFP and CRN-Cerulean) they were exported from the ER and localized to the PM. In this case, and when focusing on the PM, the signal was homogenous along the PM and overlapped with the signal coming from FM4-64 (Figs 4A, S5A, 5A, S6A). In the ER focal plane some signal could still be detected from the ER region (likely coming from newly synthesized protein) and also from small mobile structures or not nearer defined structures, which could be part of the Golgi apparatus. This signal probably came from newly synthesized protein. The pattern was, however, different from the distinct net-like structure exhibited by the ER marker (Figs 4A′, S5A′, 5A′, S6A′). For the interaction studies we used the apparent FRET between the wild-type CRN and CLV2 proteins (tagged with GFP and mCherry, respectively) as a reference value. As a negative control, we measured FRET between CLV2-mCherry and a GFP-tagged variant of CRN, in which the interaction-mediating TMD was exchanged with the non-interacting BAK1 TMD (CR(<>B1TM)-GFP) (Bleckmann *et al.*, 2010). Loss of the entire extracellular domain (ECD) of CRN (CRΔEC) (Fig. 1A, Supplementary Fig. S1B) does not abolish CLV2 interaction but impairs ER-to-PM export (Bleckmann *et al.*, 2010), indicating a regulatory role of the ECD. As there are no sequence motifs with known functions in the ECD, we further subdivided it into a N-terminal, distal part (deleted in CRΔEC1) and a proximal part adjacent to the TMD (deleted in CRΔEC2) (Fig. 1D, E, Supplementary Fig. S1B) to identify the sequences regulating export. In our CLV2 interaction assay, CRΔEC1 and CRΔEC2 exhibited apparent FRET efficiencies comparable to the wild-type CRN protein (~10%), while the efficiency for CRΔEC was ~7% (significantly different from the wild-type, Table 2). Furthermore, the CRΔEC variant was not completely exported from the ER in the presence of CLV2 (Fig. 4B, B′, Supplementary Fig. S5B, B′), while both CRΔEC1 and CRΔEC2 localized to the PM (Fig. 4C, D′, Supplementary Fig. S5C, D′). From this experiment it appears that the ECD plays a role in stabilizing the TMD-mediated interaction with CLV2. We then investigated the potential role of the amino acid motif RxR in the juxtamembrane domain of the CLV2 protein (Fig. 6A, Supplementary Fig. S1I). In the absence of CRN, CLV2 is retained in the ER, and a similar scenario has been reported for the GABA_B_ receptor, where an RxR motif actively prevents the export of the receptor from the ER (Pagano *et al.*, 2001). Interaction with CRN could be required to disguise this RxR motif and thereby allow ER export. To test if the RxR motif in the CLV2 protein is responsible for ER retention, two variants of CLV2 were created, where we either replaced the first conserved arginine (R) of the RxR motif with an alanine (A) [C2(RA)] (Fig. 6B, Supplementary Fig. S1I), or deleted the entire motif [C2(708)] (Fig. 6C, Supplementary Fig. S1I). Based on the data from the GABA_B_ receptor, we expected that mutating or deleting the RxR motif would lead to constitutive (CRN-independent) export of CLV2 from the ER. However, while both variants were able to interact with CRN (Table 2) and were exported from the ER in the presence of CRN (Fig. 5B, C′, Supplementary Fig. S6B, C′), both variants were retained in the ER in the absence of CRN, indicating that the RxR motif of CLV2 is not a functional ER-retention signal (Supplementary Figs S3K, L, S4K, L).

**Table 2.** Apparent FRET efficiencies for the different interactions

**Donor**	**Acceptor**	**D-d (%**)	**St. Dev.**	**Comment**
CRN-GFP	CLV2-mCherry	10.5	2.3	Reference 1
CR(<>B1TM)-GFP	CLV2-mCherry	3.6	2.3	Negative control
CRΔEC-GFP	CLV2-mCherry	7.2	2.0	Impaired Interaction
CRΔEC1-GFP	CLV2-mCherry	11.2	1.5	Interaction
CRΔEC2-GFP	CLV2-mCherry	10.5	2.8	Interaction
crn-1-GFP	CLV2-mCherry	5.2	3.0	Impaired interaction
crn-3-GFP	CLV2-mCherry	18.3	3.9	Interaction
CR(SD)-GFP	CLV2-mCherry	12.2	3.1	Interaction
CR(SA)-GFP	CLV2-mCherry	13.5	2.9	Interaction
CRΔKi-GFP	CLV2-mCherry	24.5	6.5	Interaction
CR(<>C1Ki)-GFP	CLV2-mCherry	10.3	3.9	Interaction
CLV2-GFP	CRN-mCherry	11.7	2.2	Reference 2
C2(RA)-GFP	CRN-mCherry	14.2	2.1	Interaction
C2(708)-GFP	CRN-mCherry	11.4	2.0	Interaction
C2(CRKi)-GFP	CRN-mCherry	5.7	3.3	Impaired interaction

D-d (%)=Donor-dequenching following photobleaching of the acceptor in %. St. Dev.=Standard deviation.

If the only role of CRN in CLV3 signaling is to transport CLV2 to the PM and mediate protein interactions there, CLV2 interaction and ER-to-PM transport should be fully correlated with protein functionality. To analyze this, we tested the variants for their capacity to actively participate in CLE peptide signaling via mutant complementation assays. For this we generated stable transgenic Arabidopsis lines by transforming *crn-3* mutants with the different gene variants expressed from the strong constitutively active cauliflower mosaic virus 35S (CaMV35S) promoter. It has been shown before that constitutive expression of *CRN* and *CLV2* from CaMV35S does not alter normal plant development (Müller *et al.*, 2008). As a first indicator for mutant complementation we determined the carpel number of the transgenic lines, thereby assaying the functionality of the proteins in shoot meristem maintenance. As a second indicator we studied their role in root meristem maintenance by measuring the length of their primary roots after treatment with the CLV3 peptide. Wild-type plants develop shorter roots when CLV3 or related CLE peptides are overexpressed or added externally (Hobe *et al.*, 2003; Fiers *et al.*, 2005). In contrast, *crn* and *clv2* mutants are insensitive to this treatment (Hobe *et al.*, 2003; Fiers *et al.*, 2005; Pallakies and Simon, 2014). In both complementation assays, the CRΔEC variant failed to complement the mutant, consistent with the observed partial retention of the protein in the ER (Table 3, Fig. 2). Interestingly, while the CRΔEC1 variant was able to restore the root length phenotype, it only reduced the severity of the carpel phenotype, while the CRΔEC2 variant was able to rescue the mutant in both assays (Table 3, Fig. 2). From these findings it seems that amino acids on the extracellular side of the CRN protein contribute to CRN protein function, even though this cannot be attributed to a single motif in the proximal or distal half of the ECD, but possibly due to a combination of motifs in both halves. We then also assayed the functionality of the two CLV2 variants, [C2(RA)] and [C2(708)] (Fig. 6). These two variants fully complemented the *clv2* mutant in both the shoot and root assays, confirming that the presumed RxR motif is not active in regulating CLV2 localization or function (Table 3, Fig. 2).

**Table 3.** Results of the mutant complementation assays

**Plant line**	**Number of carpels**			**Root length**			
	**Num.**	**SE**	***P***	**cm**	**SE**	***P***	**%**
Col-*0*	2	0	-	1.04	0.02	-	100
*crn-3*	2.37	0.08	-	1.64	0.03	-	158
*clv2-gk*	2.44	0.04	-	1.77	0.04	-	170
**CRN variants expressed in *crn*:**							
CRN	2.08 *	0.04	0.001	1.04*	0.02	1.6×10^−36^	100
CRΔEC	2.54	0.04	0.77	1.91	0.07	2.9×10^−5^	184
CRΔEC1	2.21	0.06	0.09	0.72*	0.07	1.3×10^−16^	69
CRΔEC2	2.09 *	0.05	0.004	1.10*	0.04	1.6×10^−14^	106
CRΔKi	2.22	0.07	0.18	1.00*	0.03	7.2×10^−34^	96
CR(SD)	2.04 *	0.03	6.4×10^−5^	1.10*	0.03	6.8×10^−17^	106
CR(SA)	2.32	0.09	0.70	0.96*	0.08	2.9×10^−16^	92
CR(<>C1Ki)	2.87^#^	0.19	0.01	1.46	0.11	5×10^−2^	140
C2(CRKi)	2.38	0.11	0.95	1.99^#^	0.06	2×10^−8^	191
**CLV2 variants expressed in *clv2*:**							
CLV2	2 *	0.00	1.8×10^−5^	1.10*	0.06	5.1×10^−12^	106
C2(RA)	2.01 *	0.01	8.7×10^−7^	1.00*	0.03	1.2×10^−16^	96
C2(708)	2.01 *	0.01	2.5×10^−7^	0.86*	0.06	2.5×10^−8^	83
C2(CRKi)	2.54	0.06	0.4	1.02*	0.04	1.7×10^−26^	98

Num.=Average number of carpels per silique, cm=average root length in cm after growth on CLV3-containing medium, SE=Standard error, *P*=*P*-value from Student’s *t*-test. %=Root length in percent relative to wild-type. * indicates the values significantly different from the mutant value. ^#^ indicates an enhanced mutant phenotype significantly different from the mutant value.

### The CRN kinase domain is essential for protein function in the shoot, but not in the root

**Fig. 7.**
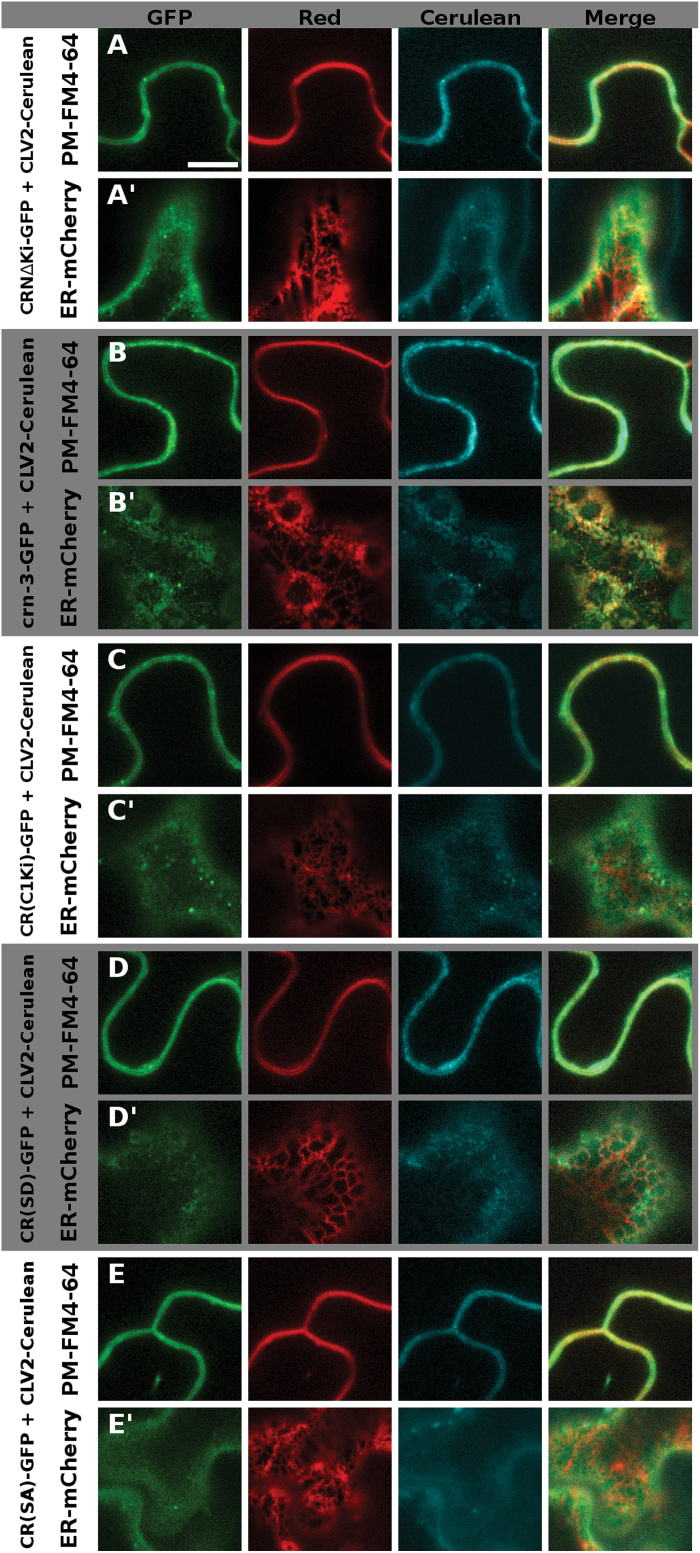
Intracellular localization of the different CRN kinase variants. The different CRN kinase variants are tagged with GFP, and CLV2 is tagged with Cerulean. In the red channel, either the membrane dye FM4-64 (A–E) or ER-localized RTNLB2-mCherry (A′-E′) is shown. CRNΔKi is shown in (A) / (A′), crn-3 in (B) / (B′), CR(C1Ki) in (C) / (C′), CR(SD) in (D) / (D′) and CR(SA) in € / (E′). In the PM focal plane (A–D), all protein variants co-localize with FM4-64 (A–E), In the ER focal plane (A′-E′), there is signal visible in all CLV2 variants tested, but different from the distinct net-like ER-structure exhibited by RTNLB2-mCherry. This net-like structure is only also visible for the crn-3 protein (B′), indicating impaired, but not completely blocked ER-export of this CRN variant. Supplementary Fig. S6 shows the same cells with switched focal planes: the FM4-64 marked cell is focused on the ER, the RTNLB2-marked cell is focused on the PM. Scale bar = 10 µm.

We then analyzed the role of the CRN kinase domain (KD). It is presumed to be catalytically inactive and not essential for the CRN protein to exert its function, which is supposed to be mediating ER-to-PM transport of CLV2 and interactions via its TMD (Bleckmann *et al.*, 2010; Nimchuk *et al.*, 2011). Interestingly, the *crn-3* mutant phenotype is caused by a truncation of the kinase, which could be taken as a first indication that the KD is necessary for full CRN protein function. To further investigate the role of the KD, we tested several protein variants for their capabilities to form complexes with CLV2, to mediate ER-to-PM transport, and for their functionality in the shoot and root mutant complementation assays. These analyses first revealed that a shortened CRN protein lacking the kinase (CRΔKi) (Fig. 1G, Supplementary Fig. S1A–D) can still interact with CLV2 via the TMD and facilitate its ER-to-PM export (Table 2, Fig. 7A, A′’, Supplementary Fig. S7A,A′) (Bleckmann *et al.*, 2010). The measured apparent FRET efficiency of 24.5% was even higher than the 10.5% for the wild-type variant, probably due to the GFP being in closer proximity to the CLV2-fused mCherry when the kinase is missing. However, the transformed mutant plants still produced siliques with 2.22 (±0.07) carpels, indicating that the CRΔKi variant cannot rescue the mutant phenotype and that the KD is necessary for CRN protein function in the floral meristem (Table 3, Fig. 2A, B). Intriguingly, the roots of the CRΔKi expressing lines were comparable in length to those of the wild-type when challenged with CLE peptides, showing that, in contrast to the shoot, the CRΔKi variant is able to restore CRN function in the root (Table 3, Fig. 2C, D). Our interpretation of this finding is that although the CRN KD is potentially inactive, it is needed for shoot meristem maintenance, either for sterical reasons, as a scaffold for the assembly of downstream signaling components, or as a substrate for phosphorylation by the activated CLV1 kinase. In the root meristem, however, CRN function could be limited to facilitate CLV2 ER export and PM localization. Interestingly, removing the entire kinase domain, as done in the CRΔKi variant, can rescue the *crn-3* root phenotype, which is caused by a truncation of this kinase. Our interpretation of this observation, that a truncated kinase causes a mutant phenotype while removing it completely does not, is that the crn-3 version of the kinase is partially unfolded or folded incorrectly, which could impair the protein’s interaction with CLV1, with other so-far unknown components of the complex, or possibly with scaffolding proteins in the ER that affect protein folding and subsequent export of the protein to the PM. Fittingly, PM localization of the crn-3 variant was not as homogeneous as it was for the wild-type protein and some co-localization with the ER-marker could still be observed in the presence of CLV2 (Fig. 7B,B′, Supplementary Fig. S7B-B’). We then asked whether CRN and CLV2 have to act as separate molecules, which would indicate that they also perform independent functions, or if a fusion protein of CLV2 with CRN can actively signal. We expressed a chimeric protein C2(CRKi) that consisted of the CLV2 LRR-receptor and its TMD fused to the CRN KD close to the juxtamembrane domain (JD) (Fig. 6D, Supplementary Fig. S1E, G–I). This chimeric protein is retained in the ER when expressed without CRN, showing that the presence of the CRN kinase domain at the CLV2 protein is not sufficient to mediate export from the ER to the PM (Supplementary Figs S3M, S4M). When co-expressed, the CRN protein was still able to interact with the C2(CRKi) chimera and mediated its export from the ER, even though both interaction and export seemed to be impaired (Table 2, Fig. 6D, D′). We then tested the protein’s ability to complement the *crn* or *clv2* mutant phenotypes in shoot and root. As expected from the retention of C2(CRKi) chimera in the ER in the absence of CRN, it could not rescue the *crn* mutant in either the shoot or the root assay (Table 3, Fig. 2). When expressed in the *clv2* mutant background, however, the C2(CRKi) protein completely restored the root phenotype, but failed to rescue the carpel phenotype (Table 3, Fig. 2). This could imply that the additional kinase domain, attached to the CLV2 protein, might impair the function of the endogenous CRN kinase, for example by impeding interaction between CLV2 and CRN or with another RLK, or by the binding of downstream components to the kinase domain. Accordingly, as it was the case for the CRΔKi variant, it appears that the CRN kinase domain is necessary for SAM maintenance but not root meristem maintenance. One possible interpretation of these results is that the CRN kinase mediates interactions with downstream effector proteins of CLV signaling in the shoot. For this, the KD does not have to be catalytically active. We therefore asked if the CRN kinase domain can be functionally replaced by another kinase domain. To test this, we constructed a second chimera in which we replaced the presumed inactive kinase of CRN with the catalytically active kinase domain of CLV1 [CR(<>C1Ki)] (Fig. 1I, SupplementaryFig. S1A–D, F). If CRN has a specific set of downstream effector proteins, the CLV1 kinase should not be able to take over CRN function. The CR(<>C1Ki) chimera interacted with CLV2 and was exported from the ER (Table 2, Fig. 7C, C′’, Supplementary Fig. S7C, C′), but it did not rescue the *crn* mutant in either complementation assay (Table 3, Fig. 2). Interestingly, the carpel phenotype was even enhanced in the CR(<>C1Ki) transgenic lines, which indicates a dominant negative effect of the chimeric protein (Fig. 2A, B). From these observations it appears that the CLV1 kinase cannot substitute for the CRN kinase, indicating that the CLV1 and CLV2/CRN complexes act in different fashions. To further explore whether the CRN kinase is actively involved in signaling we created putative phosphomimetic and phosphomute variants [CR(SD) and CR(SA)] of the CRN kinase. A potential phosphorylation site has been described previously at serine 156 (Nühse *et al.*, 2004; Zulawski *et al.*, 2013). By replacing this serine with an aspartic acid (S156D), which is structurally similar to a phosphoserine, we aimed at creating a constitutively active variant of the protein (Fig. 1H, Supplementary Fig. S1E). By replacing S156 with an alanine (S156A) we aimed at creating a constitutively inactive variant (Fig. 1H, Supplementary Fig. S1E). These two variants both interacted with CLV2 and facilitated CLV2 ER export (Table 2, Fig. 7D, E′, Supplementary Fig. S7D, E′). However, only the CR(SD) variant complemented the *crn-3* mutant in both carpel and root assay (Table 3, Fig. 2), while the CR(SA) variant was only able to complement the root, but not the carpel phenotype (Table 3, Fig. 2). This again points to a role for the CRN kinase in shoot meristem, but not proximal root meristem maintenance. We therefore conclude that although the CRN kinase domain does not show catalytic activity, it could be a target for phosphorylation and mediate the interaction with other kinases.

Taken together these results strongly support the notion that CRN is not solely a scaffolding protein for CLV2, but specifically contributes to CLV3 signaling via its pseudokinase domain. Furthermore, it appears to function in a different manner than the CLV1 KD, potentially through a different set of downstream effector proteins.

## Discussion

With this work we tried to answer the question whether the CRN protein is more likely a scaffolding protein, necessary to localize CLV2 to the PM and mediate interaction between CLV2 and CLV1, or if it is also involved in transmitting the CLV3 signal via its kinase domain. For the first part we focused on the ECD of CRN and the juxtamembrane domain of CLV2. We found that the extracellular domain of the CRN protein seems to play a stabilizing role for the interaction between CRN/CLV2. Removing the entire ECD domain results in impaired CLV2 interaction and ER export. The deletion of only parts of the domain does not affect the protein’s interaction with CLV2 or its localization. Interestingly, it does not matter if the distal or more proximal part of the domain is deleted in this regard. However, only the variant lacking the proximal half can complement the *crn* mutant phenotype in both the root and the shoot meristem, while the variant lacking the distal part can only complement the root length phenotype. Therefore, it can be assumed that parts of the ECD are necessary to stabilize the CRN/CLV2 complex for ER export. Once in a complex with CLV2 and localized to the cell’s PM, combinations of motifs in the distal and proximal half of the ECD could also be important, e.g. to support conformational changes in the extracellular receptor domain of the CLV2 protein. The CLV2 variants, in which a potential RxR motif is mutated [C2(RA) and C2(708)], were still retained in the ER in the absence of the CRN protein: if this was an actual ER-retention signal, as it has been determined for the GABA_B_ receptor, they should have been constitutively exported. Since both variants were also able to complement the *clv2* mutant, it appears that the engineered mutations had no effect. Therefore, the presumed motif does not appear to be a functional RxR motif.

**Fig. 8.**
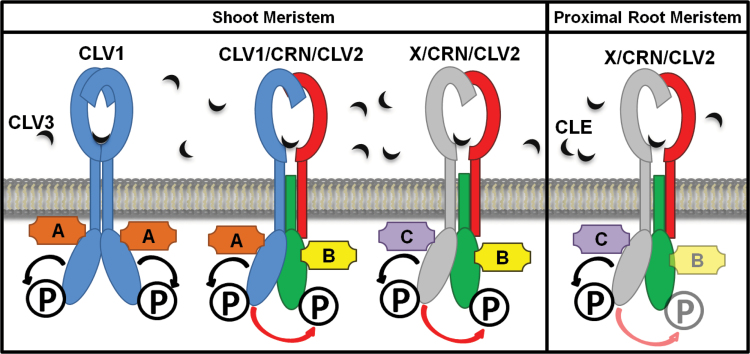
Model of the different receptor complexes involved in CLE signaling in the shoot and proximal root meristem. In the shoot, two CLV1 proteins (blue) form signaling-active homomers that can bind CLV3 (black) via their receptor domains and autophosphorylate (black arrows and ‘P’) on their kinase domains. The CLV2 (red) / CRN (green) heteromers interact either with CLV1 or an unknown receptor ‘X’ (light grey) to form multimers. The interacting RLKs may bind CLV3 together with CLV2, and transphosphorylate (red arrows) the CRN kinase domain. Following phosphorylation of the kinases, different effector proteins interact with the kinase domains of either CLV1 (‘A’, orange) or CRN (‘B’, yellow) and Protein ‘X’ (‘C’, purple). In the proximal root meristem the CLV2 (red) / CRN (green) heteromers function independently of CLV1, but possibly together with an unknown receptor ‘X’ (light grey). Here, receptor ‘X’ perceives a CLE peptide signal together with CLV2. Downstream signaling is independent of the CRN kinase, possibly due to autophosphorylation of the kinase domain of receptor ‘X’ and its effector protein ‘C’ (purple).

We then focused on elucidating the functionality of the presumed CRN pseudokinase domain. The first important finding is that CRN appears to function in different fashions in the two CLE peptide-dependent stem cell maintenance pathways in the shoot and the root meristem (Fig. 8). Furthermore, we show that CRN–CLV2 interaction and ER-to-PM transport of the proteins is not sufficient to complement the *crn* mutant: out of all the kinase variants we analyzed, all showed interaction with CLV2 and were transported to the PM. However, only a few were able to complement the mutant and restore the phenotype to the wild-type. According to this finding it is unlikely that CRN is only a scaffolding protein, or solely required to localize CLV2 to the PM: more likely it is actively involved in transmitting the CLE peptide signal.

In root meristem maintenance, the CRN protein appears to be functional even without its kinase domain (CRΔKi). While this finding is in line with the CRN kinase being catalytically inactive, it does leave the question how the CRN/CLV2 complex relays the CLE peptide signal into the cells of the proximal root meristem since the intracellular CRN kinase is dispensable. One possibility is that the proteins act in a complex with another, so far unidentified RLK (‘X’ in Fig. 8). This RLK cannot be CLV1, since the expression pattern of *CLV1* in the root meristem does not overlap with the combined pattern of *CRN* and *CLV2*, and *clv1* mutants are still sensitive to externally added or overexpressed CLE peptides (Hobe *et al.*, 2003; Fiers *et al.*, 2005; Stahl *et al.*, 2013; Pallakies and Simon, 2014). Nevertheless, although the kinase is not necessary for CRN function, the mutant phenotype of *crn-3* plants is caused by a truncation of the kinase. Thus, we can conclude that the truncated kinase interferes with the formation of a larger complex, which could involve RECEPTOR-LIKE PROTEIN KINASE 2 (RPK2) or BARELY ANY MERISTEM 1 (BAM1) (Betsuyaku *et al.*, 2011; Shimizu *et al.*, 2015).

In the shoot meristem, CRN actively participates in CLV3 signaling through its kinase domain. A CRN variant that lacks the kinase domain (CRΔKi) is unable to complement the *crn* carpel phenotype. Introducing a second copy of the kinase by expressing a chimeric protein of CLV2 with the CRN kinase fused to it cannot rescue the mutant either, indicating that the additional kinase in the CRN/CLV2 complex either hinders the endogenous CRN kinase sterically or competes with it for downstream effector proteins. Interestingly, replacing the CRN kinase domain with that of CLV1 [CR(<>C1Ki)] did not restore CRN function in the shoot of *crn* mutants either, but generated a dominant negative effect. This indicates that the CLV1 and CRN kinase domains act differently and signal via their own specific sets of downstream effector proteins. The additional copy of the CLV1 kinase, attached to the CRN protein, may compete with the native CLV1 kinase for its specific interactors. Interestingly, the only CRN kinase variant that rescued the *crn* mutant shoot phenotype was the potential phosphomimetic version [CR(SD)]. In this variant the KD is supposed to phenocopy a constitutively phosphorylated kinase. The counterpart, a phosphomute variant of the CRN kinase, where the potential phosphorylation site was replaced by an alanine [CR(SA)], was unable to rescue the carpel phenotype. Therefore, we hypothesize that CLV3 or other CLE peptide binding induces transphosphorylation of the CRN kinase domain, either by CLV1 or by another unknown RLK. This phosphorylated CRN kinase domain could then transmit the CLE signal to downstream effector proteins., CRN has consistently been shown previously to be phosphorylated at S156 (Nühse *et al.*, 2004; Zulawski *et al.*, 2013; note that S156 is sometimes referred to as S131 in other work: this is due to the authors mapping it to gene model *AT5G13290.1*, which represents a shorter splicing variant, spliced in less than 3% of all *CRN* mRNAs; Müller *et al.*, 2008).

While most work on pseudokinases has been performed in mammalian cells (Boudeau *et al.*, 2006; Kwak *et al.*, 2014), a role for pseudokinases has also emerged in plants: in the kinase domain of the STRUBBELIG (SUB)/SCRAMBLED (SCM) RLK, several essential amino acids of the catalytic loop are not conserved, rendering it inactive (Chevalier *et al.*, 2005). However, a mutant variant of the kinase, *sub-4*, which carries a point mutation in a conserved arginine in kinase subdomain VIa, was shown to cause a mutant phenotype. Furthermore, Kwak *et al.* (2014) could assign the function of the SUB kinase in different signaling processes to different specific residues within the kinase domain, showing that the kinase domain is important for protein functionality. In plant immune signaling, the inactive pseudokinase BAK1-interacting receptor-like kinase 2 (BIR2) has been shown to be transphosphorylated on several residues in its juxtamembrane domain by the interacting kinase BAK1, indicating that BIR2 functions as a kinase substrate and contributes to signaling by mediating effector protein interactions (Halter *et al.*, 2014).

From our data, we propose two partially distinct modes of action for CRN in shoot and root meristems (Fig. 8). Stem cell fate in the shoot is controlled by CLV1, CLV2, CRN, and several other kinases, which could include RPK2 and BAM RLKs. CLV1 homomers are activated by CLV3, resulting in autophosphorylation and ensuing interaction with downstream effectors (‘A’ in Fig. 8). In addition, heteromeric complexes consisting of CLV1, CLV2, and CRN form that bind CLV3. CLV1 activation would cause autophosphorylation and also phosphorylation of the inactive CRN kinase within the complex. This would then again trigger interaction with effector ‘A’, while the CRN kinase might signal through a different effector (‘B’ in Fig. 8). CLV1-independent signaling of the CRN/CLV2 pathway is achieved through its interaction with an unknown RLK (‘X’ in Fig. 8), which autophosphorylates and acts through effector ‘C’, but also activates the CRN KD. In the proximal root meristem, *CLV1* is not expressed. Here, the RLK ‘X’ is the sole partner for the CRN/CLV2 complex. The CRN KD is now not required, indicating that effector ‘C’ suffices for root meristem homeostasis.

## Supplementary data

Supplementary data are available at *JXB* online.

Figure S1. Amino acid sequence of the different CRN and CLV2 protein domains.

Figure S2. Complementation of the *crn-1* and *clv2-1* carpel phenotype.

Figure S3. Intracellular localization of the different CRN and CLV2 variants without the co-expressed partner, with the focal plane showing the PM.

Figure S4. Intracellular localization of the different CRN and CLV2 variants without the co-expressed partner, with the focal plane showing the ER.

Figure S5. Intracellular localization of the different CRN ECD variants.

Figure S6. Intracellular localization of the different CLV2 variants.

Figure S7. Intracellular localization of the different CRN kinase variants.

Supplementary Data
