# Supplementary material for: Shared and distinct functions of the pseudokinase CORYNE (CRN) in shoot and root stem cell maintenance of Arabidopsis
Source: J Exp Bot. 2016 May 26;67(16):4901–15. doi: 10.1093/jxb/erw207 (PMC4983110; doi:10.1093/jxb/erw207)
Supplement: Supplementary Data [file supp_67_16_4901__index.html]

Shared and distinct functions of the pseudokinase CORYNE (CRN) in shoot and root stem cell maintenance of Arabidopsis — Shared and distinct functions of the pseudokinase CORYNE (CRN) in shoot and root stem cell maintenance of Arabidopsis — Supplementary Data 

# Shared and distinct functions of the pseudokinase CORYNE (CRN) in shoot and root stem cell maintenance of Arabidopsis

## Supplementary Data

Data files

- supplementary\_figures\_S1\_S7.pdf - Supplementary Data
