# Supplementary material for: Shared and distinct functions of the pseudokinase CORYNE (CRN) in shoot and root stem cell maintenance of Arabidopsis
Source: J Exp Bot. 2016 May 26;67(16):4901–15. doi: 10.1093/jxb/erw207 (PMC4983110; doi:10.1093/jxb/erw207)
Supplement: Supplementary Data [file supp_erw207_supplementary_figures_S1_S7.pdf]

Supplemental Information:

CORYNE:

A) **SP:**
MKQRRRRNGCSSSNTISLLLLFFLVFFSRTSTS

B) **EC:**
TS*CRRRTVKHLS**TTSTSSTPLESRI**TSK*

C) **TMD:**
VIVISIVSGILTGLVSALVLAFL

D) **JD:**
VRSIVKFMK

E) **KD:**
QTPILKGPVVFSPKITPKSLHAALSNGIQLLGSDLNGKYYKMVLDNGLVVAVKRLGSLEGVG**S**PESSSSKSV
KRRLQKELELLAGLRHRNLMSLRAYVRESDEFSLVYDYMPNGSLEDVMNKVRTKEVELGWEIRLRVAVGI
VKGLQYLHFSCETQILHYNLKPTNVMLDSEFEPRLADCGLAKIMPSSHTAVSCYSAPESSQSNRYTDKSDIF
SFGMILGVLLTGRDPTHPFCEESASGGSLGQWLKHLQQSGEAREALDKTILGEEVEEDEMLMALRITIICLS
DFPADRPSSDELVHMLTQLHSF

F) **CLV1-KD:**
KKNQKSLAWKLTAFQKLDFKSEDVLECLKEENIIGKGGAGIVYRGSMPNNVDVAIKRLVGRGTGRSDHGF
TAEIQTLGRIRHRHIVRLLGYVANKDTNLLLYEYMPNGSLGELLHGSKGGHLQWETRHRVAVEAAKGLCYL
HHDCSPLILHRDVKSNNILLDSDFEAHVADFGLAKFLVDGAASECMSSIAGSYGYIAPEYAYTLKVDEKSDV
YSFGVVLLELIAGKKPVGEFGEGVDIVRWVRNTEEEITQPSDAAIVVAIVDPRLTGYPLTSVIHVFKIAMMC
VEEEAAARPTMREVVHMLTNPPKSVANLIAF

CLAVATA2:

G) **LRR:**
LPDLDPQDKASLLIFRVSIHDLNRSLSTWYGSSCSNWTGLACQNPTGKVLSLTLSGLNLSSQIHPSLCKLSSL
QSLDLSHNNFSGNIPSCFGSLRNLRTLNLSRNRFVGSIPATFVSLKELREVVLSENRDLGGVVPHWFGNFS
MNLERVDFSFCSFVGELPESLLYLKSLKYLNLESNNMTGTLRDFQQPLVVLNLASNQFSGTLPCFYASRPSL
SILNIAENSLVGGLPSCLGSLKELSHLNLSFNGFNYEISPRLMFSEKLVMLDLSHNGFSGRLPSRISETTEKLGL
VLLDLSHNSFSGDIPLRITELKSLQALRLSHNLLTGDIPARIGNLTYLQVIDLSHNALTGSIPLNIVGCFQLLAL
MISNNNLSGEIQPELDALDSLKILDISNNHISGEIPLTLAGLKSLEIVDISSNNLSGNLNEAITKWSNLKYLSLA
RNKFSGTLPSWLFKFDKIQMIDYSSNRFSWFIPDDNLNSTRFKDFQTGGGEGFAEPPGKVEIKISAAVVAK
DELSFSYNLLSMVGIDLSDNLLHGEIPEALFRQKNIEYLNLSYNFLEGQLPRLEKLPRLKALDLSHNSLSGQVI
GNISAPPGLTLLNLSHNCFSGIITEKEGLGKFPGALAGNPELCVETPGSKCDPANIDASQEEIYQNELVEGP

H) **TMD:**
ISIWIFCLSAFISFDFG

I) **JD:**
VLGIFCSA***RAR**SYILQTKA*

Figure S1: Amino acid sequence of the different CRN and CLV2 protein domains

A) Signal Peptide of the CRN protein. B) Extracellular domain (ECD) of CRN. Amino acids (aa) in italic were removed in the CR Δ EC variant. For the CR Δ EC1 variant, the underlined sequence was deleted, for the CR Δ EC2 variant the aa printed in bold were deleted. C) Transmembrane domain (TMD) of CRN. The underlined G is replaced by an E in the crn-1 mutant. D) Juxtamembrane domain (JD) of CRN. E) Kinase domain

(KD) of CRN. In the CR Δ Ki variant, it is removed. The crn-3 mutant variant contains only the underlined aa. For the phosphomimetic (CRN(SD)) / phosphomute (CR(SA)) variants the bold S was replaced by either a D or an A. To create the C2(CRKi) chimeric variant, this sequence was added to the CLV2 sequence of G, H and I. F) The kinase domain of CLV1. In the CR(<>C1Ki) variant, the CRN KD (E) was replaced with this sequence from CLV1. G) The LRR-receptor domain of CLV2. H) The TMD of CLV2. I) The juxtamembrane domain of CLV2. The putative RxR motif is printed in bold. In the C2(RA) variant, the underlined R was replaced by an A. For the C2(708) variant, the aa in italic were removed.

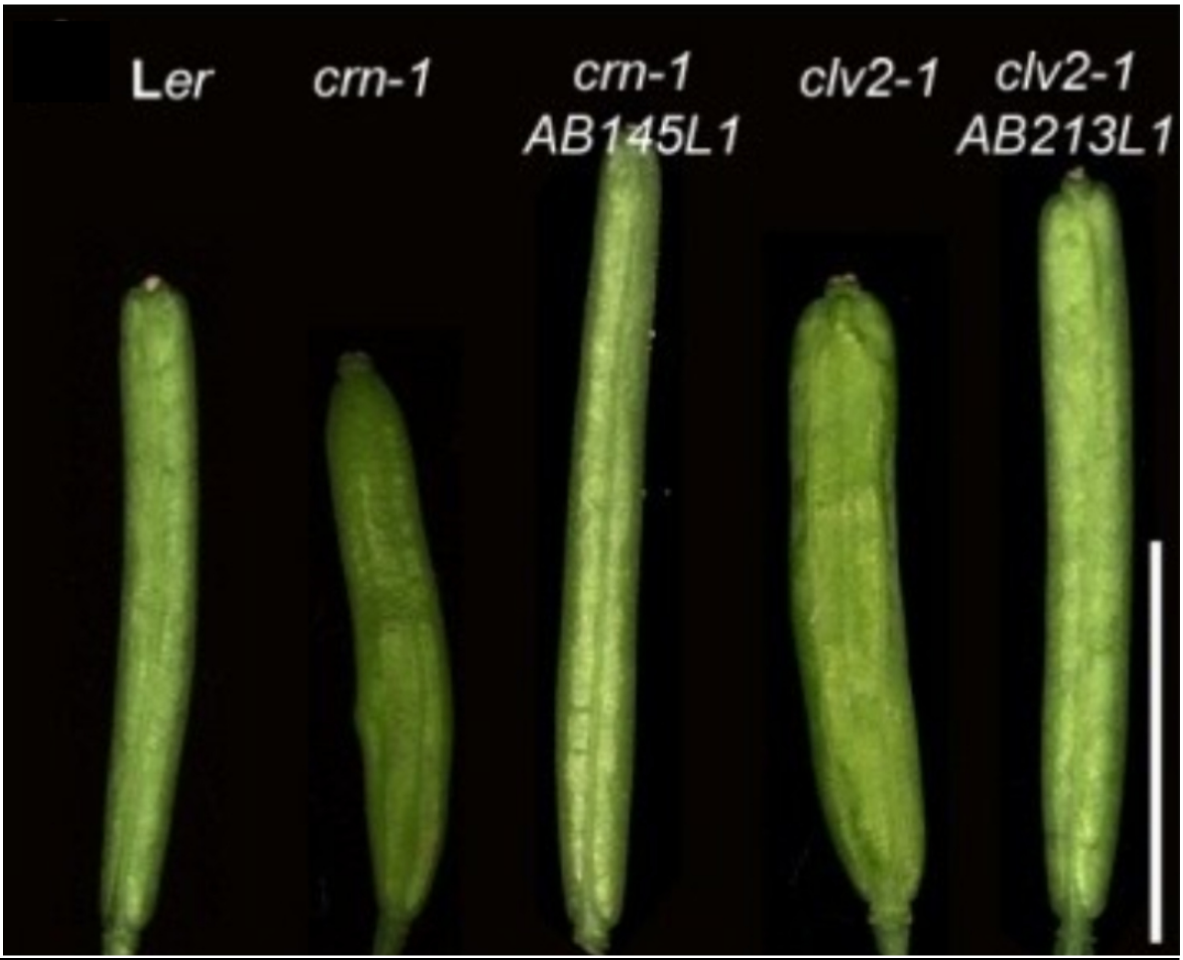

<u>Figure S2:</u> Complementation of the *crn-1* and *clv2-1* carpel phenotype

Carpel phenotypes of the Landsberg *erecta* wild type (L*er*) and the *crn-1* and *clv2-1* mutants. The *crn-1* mutant phenotype could be rescued by introducing a *CRN::CRN-GFP* (*AB145L1*) transgene, the *clv2-1* phenotype by a *CLV2::CLV2-GFP* (*AB213L1*) transgene. For the *crn-1 AB145* construct, four independent transgenic lines were obtained, which all restored the carpel number per silique (Student t-test with $p < 0.005$.). For the *clv2-1 AB213* construct, twenty independent transgenic lines were obtained, which all restored the carpel number per silique (Student t-test with $p < 0.005$.). Scale bar = 1 cm.

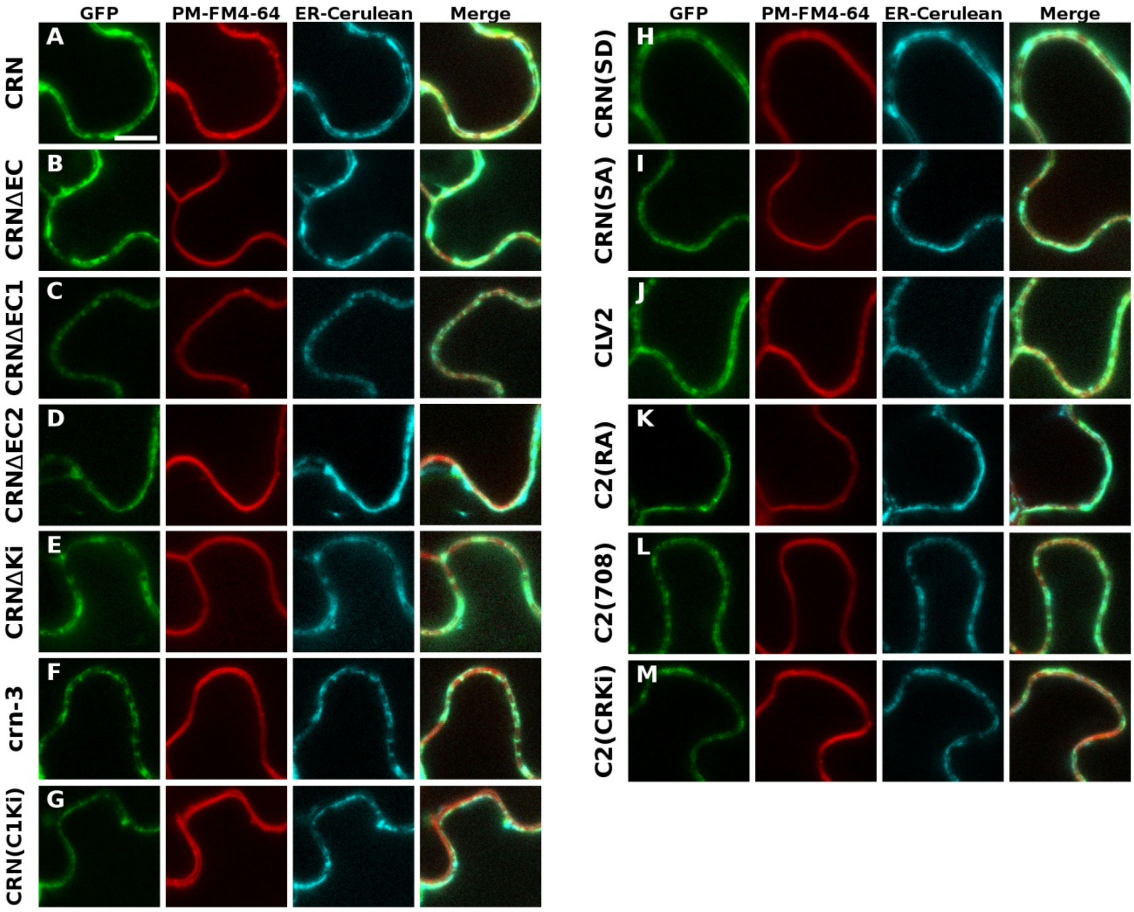

<u>Figure. S3:</u> Intracellular localization of the different CRN and CLV2 variants without co-expressed partner

The focal-plane was chosen to show the PM (Figure S3 shows the ER-focal-plane of the same cell). The different CRN and CLV2 kinase variants are tagged with GFP (GFP), the membrane is stained by FM4-64 (PM-FM4-64) and the ER is marked by RTNLB2-Cerulean (ER-Cerulean). All variants co-localize with RTNLB2-Cerulean in the ER, displaying a patchy pattern along the PM. Scale bar = 10 µM.

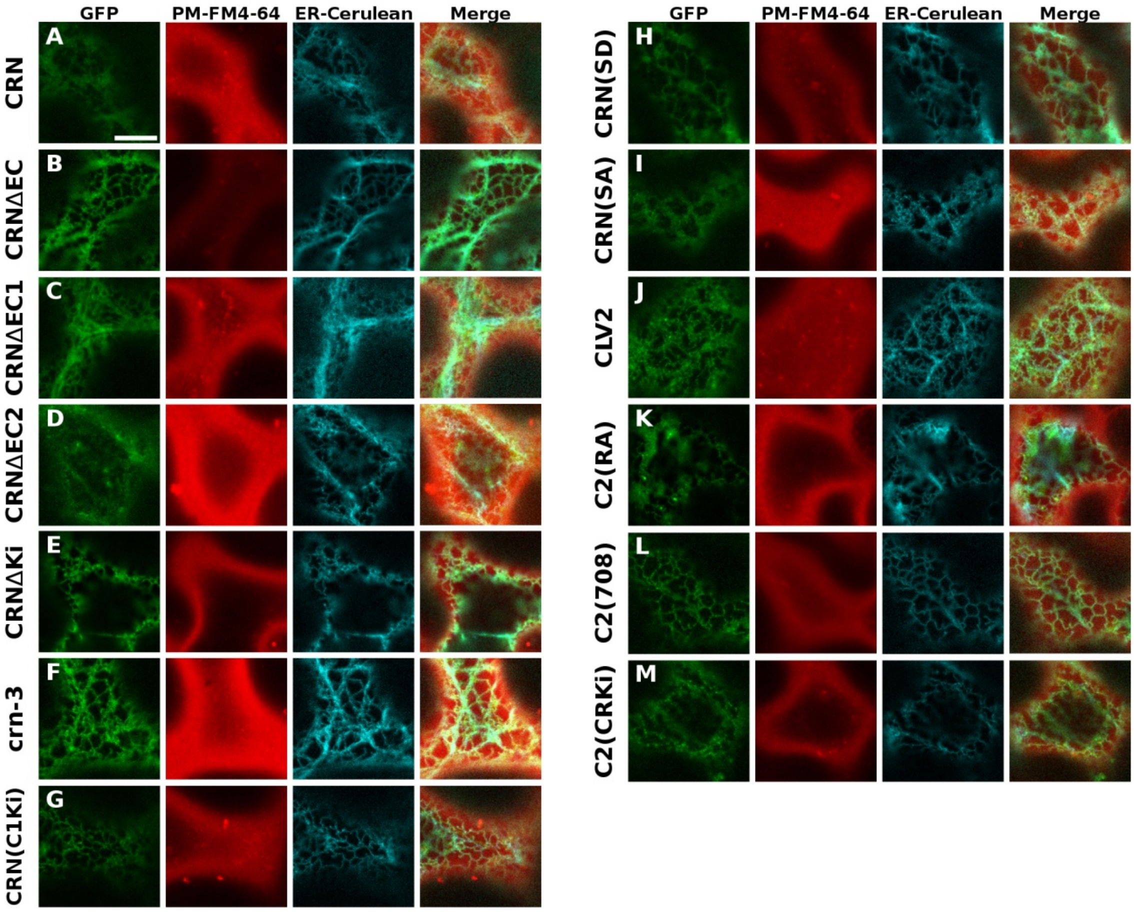

<u>Figure S4:</u> Intracellular localization of the different CRN and CLV2 variants without co-expressed partner

The focal-plane was chosen to show the ER (Figure S2 shows the PM-focal-plane of the same cell). The different CRN and CLV2 kinase variants are tagged with GFP, the membrane is stained by FM4-64 (PM-FM4-64) and the ER is marked by RTNLB2-Cerulean (ER-Cerulean). All variants co-localize with RTNLB2-Cerulean in the ER. Scale bar = 10 µM.

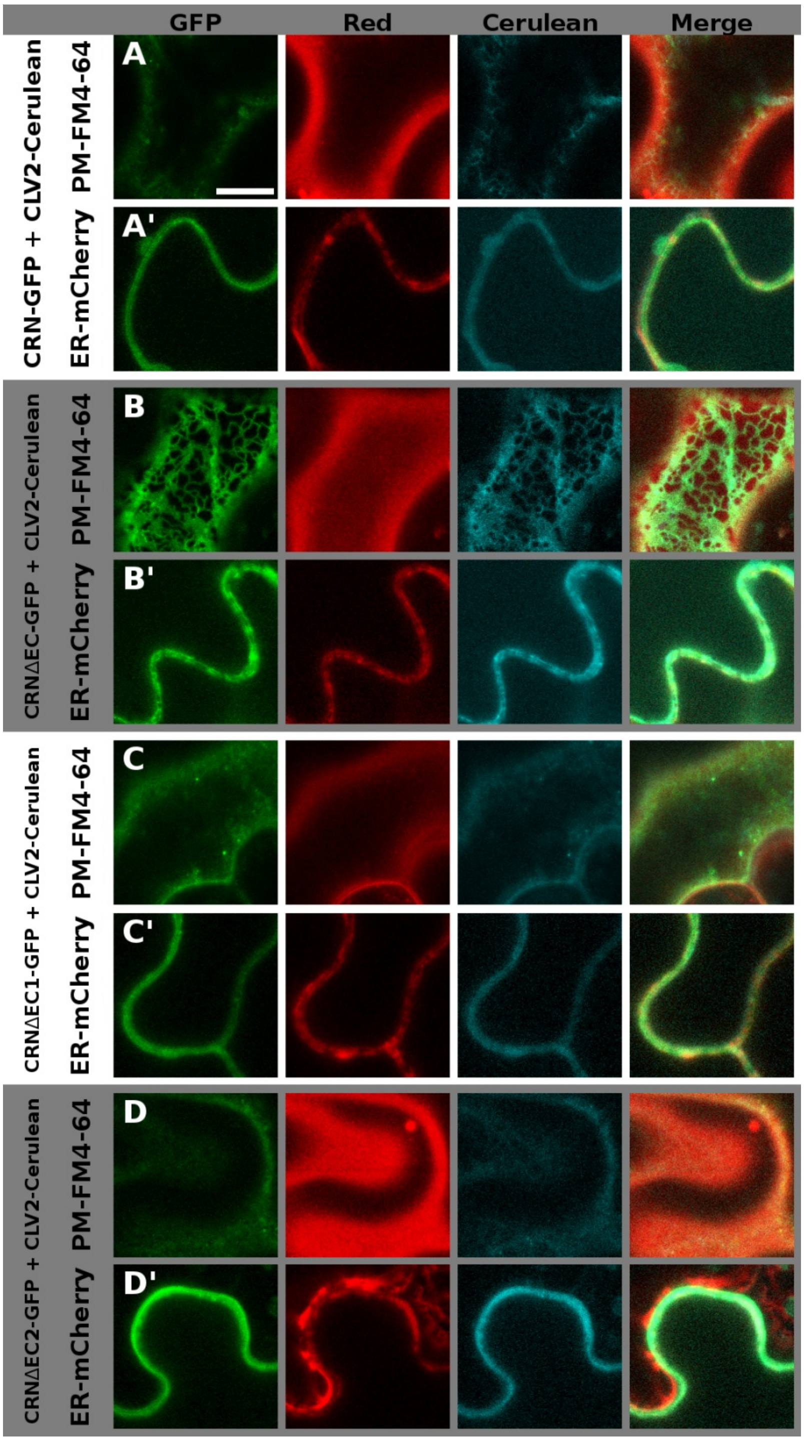

Figure S5: Intracellular localization of the different CRN ECD variants

Counter-picture to Figure 4: The focal-plane was chosen to show the ER in the FM4-64 marked cells (A-D) and the PM in the RTNLB2-marked cell (A'-D'). Scale bar = 10 µM.

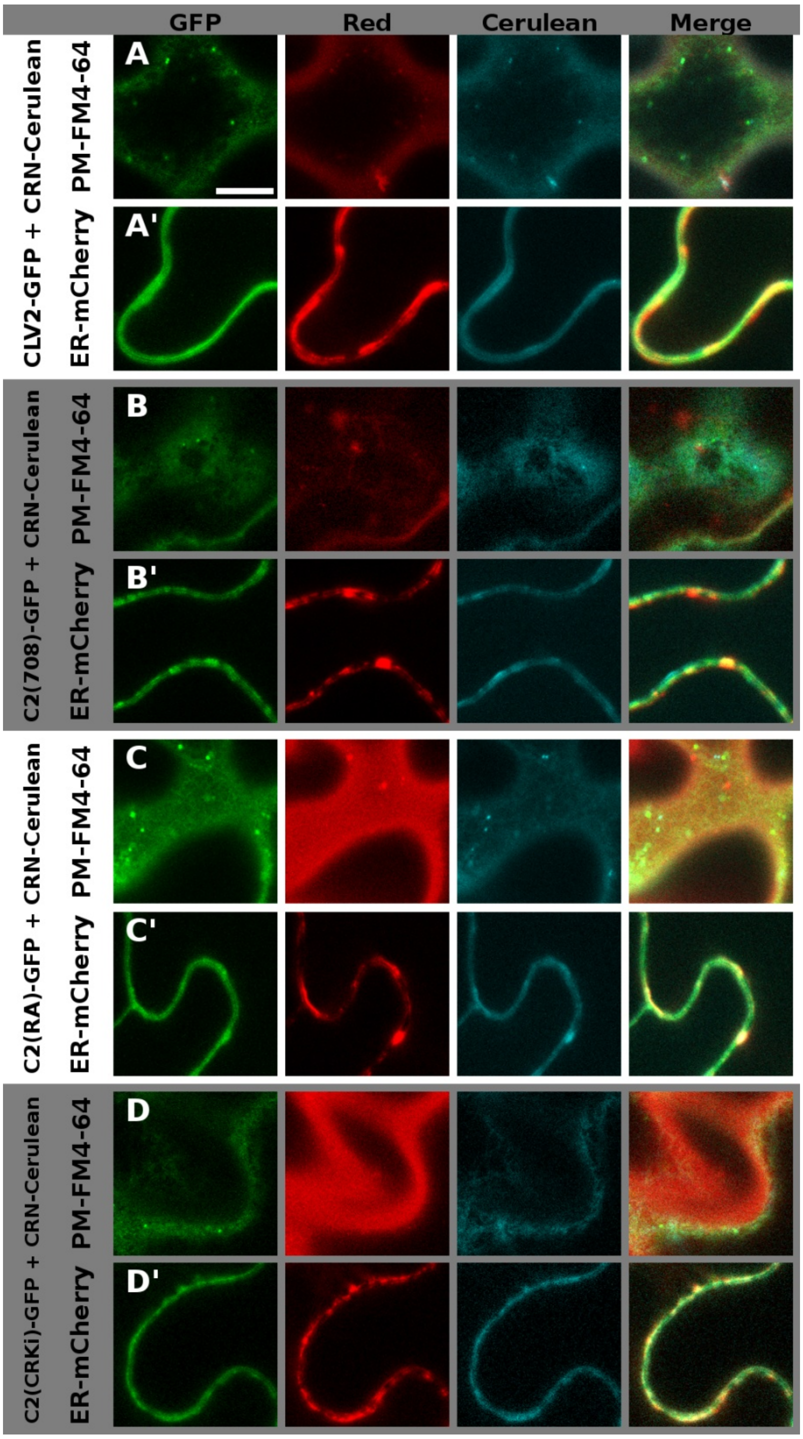

<u>Figure S6:</u> Intracellular localization of the different CLV2 variants

Counter-picture to Figure 6: The focal-plane was chosen to show the ER in the FM4-64 marked cells (A-D) and the PM in the RTNLB2-marked cell (A'-D'). Scale bar = 10 µM.

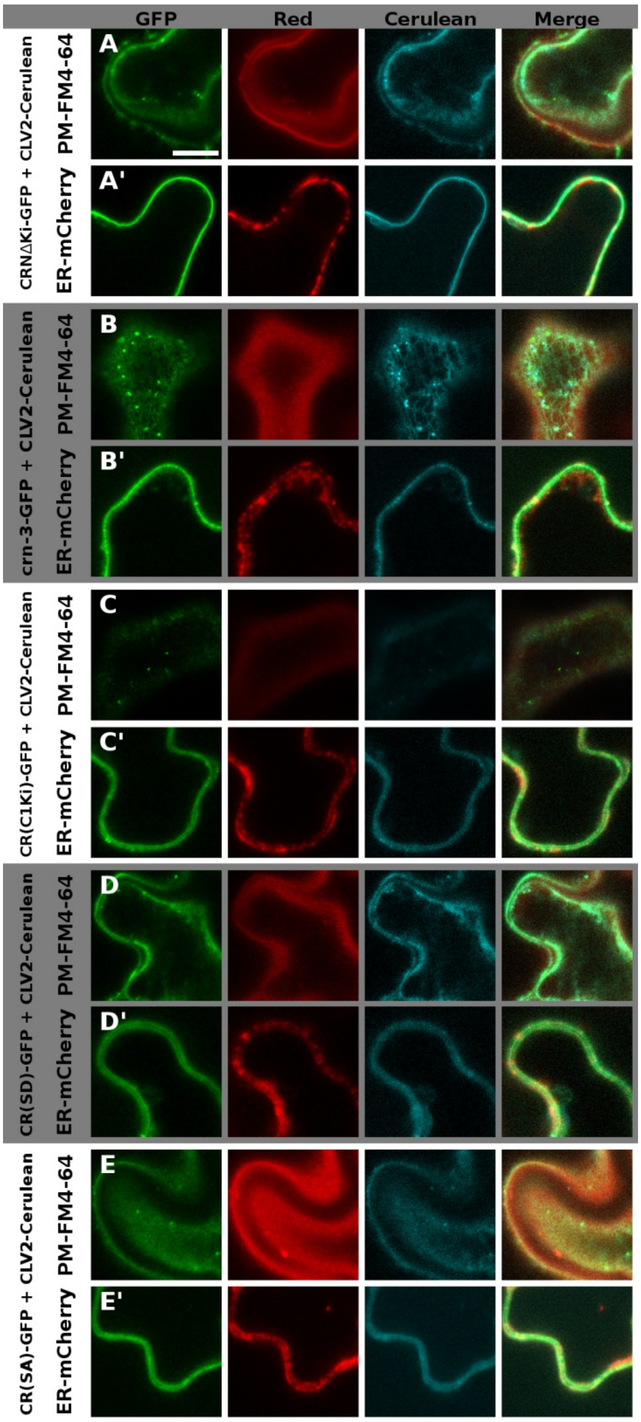

<u>Figure S7:</u> Intracellular localization of the different CRN kinase variants

Counter-picture to Figure 7: The focal-plane was chosen to show the ER in the FM4-64 marked cells (A-D) and the PM in the RTNLB2-marked cell (A'-D'). Scale bar = 10 μM.
